# Molecular imaging with ^99m^Tc-MIBI and molecular testing for mutations in differentiating benign from malignant follicular neoplasm: a prospective comparison

**DOI:** 10.1007/s00259-015-3285-1

**Published:** 2015-12-23

**Authors:** L. Giovanella, A. Campenni, G. Treglia, F. A. Verburg, P. Trimboli, L. Ceriani, M. Bongiovanni

**Affiliations:** Department of Nuclear Medicine and Thyroid Centre, Oncology Institute of Southern Switzerland, Via Ospedale 12, 6500 Bellinzona, Switzerland; Policlinico Universitario, Istituto di Medicina Nucleare, Messina, Italy; Department of Nuclear Medicine, RWTH University Hospital Aachen, Aachen, Germany; Ospedale Israelitico, Sezione di Endocrinologia e Diabetologia, Roma, Italy; Centre Hopitalier Universitaire Vaudouise, Institut de Pathologie, Lausanne, Switzerland

**Keywords:** Thyroid, Follicular neoplasms, Fine-needle aspiration cytology, ^99m^Tc-MIBI, Molecular markers, Scintigraphy, Mutation analysis

## Abstract

**Purpose:**

To compare mutation analysis of cytology specimens and ^99m^Tc-MIBI thyroid scintigraphy for differentiating benign from malignant thyroid nodules in patients with a cytological reading of follicular neoplasm.

**Methods:**

Patients ≥18 years of age with a solitary hypofunctioning thyroid nodule (≥10 mm), normal thyrotropin and calcitonin levels, and a cytological diagnosis of follicular neoplasm were prospectively enrolled. Mutation analysis and ^99m^Tc-MIBI scintigraphy were performed and patients were subsequently operated on to confirm or exclude a malignant lesion. Mutations for *KRAS*, *HRAS* and *NRAS* and for *BRAF* and translocations of PAX8/PPARγ, RET/PTC1 and RET/PTC3 were investigated. Static thyroid scintigraphic images were acquired 10 and 60 min after intravenous injection of 200 MBq of ^99m^Tc-MIBI and visually assessed. Additionally, the MIBI washout index was calculated using a semiquantitative method.

**Results:**

In our series, 26 % of nodules with a follicular pattern on cytology were malignant with a prevalence of follicular carcinomas. ^99m^Tc-MIBI scintigraphy was found to be significantly more accurate (positive likelihood ratio 4.56 for visual assessment and 12.35 for semiquantitative assessment) than mutation analysis (positive likelihood ratio 1.74). A negative ^99m^Tc-MIBI scan reliably excluded malignancy.

**Conclusion:**

In patients with a thyroid nodule cytologically diagnosed as a follicular proliferation, semiquantitative analysis of ^99m^Tc-MIBI scintigraphy should be the preferred method for differentiating benign from malignant nodules. It is superior to molecular testing for the presence of differentiated thyroid cancer-associated mutations in fine-needle aspiration cytology sample material.

## Introduction

The diagnostic approach to thyroid nodules is usually based on clinical examination, ultrasonography (US) and scintigraphy in suspicious nodules followed by the well-established complementary procedure of fine-needle aspiration cytology (FNAC) [1]. However, the most important limitation of FNAC is the lack of accuracy in discriminating between malignant follicular carcinoma and benign follicular adenoma because of its inability to detect capsular invasion and vascular infiltration of the tumour [2]. An additional limitation is the fact that there are some patients with follicular variants of papillary carcinoma in whom the classic diagnostic cytological criteria of papillary carcinoma are lacking (i.e. papillary structures) or not completely unequivocal (i.e. nuclear features) [3]. Furthermore, in some patients with a microfollicular goitre with a hypercellular pattern, diagnosis by FNAC may be a challenge [4]. As a consequence, any nodule diagnosed as a follicular neoplasia on FNAC requires surgical excision for definitive histopathological diagnosis [2, 4]. However, the great majority of such cases turn out to be histologically benign. Hence, the identification of new diagnostic approaches to provide reliable preoperative criteria for malignancy in patients with indeterminate FNAC is of paramount importance to reduce the number of unnecessary thyroid surgery procedures. Different additional tools have been proposed to increase preoperative accuracy in the assessment of nodules with a follicular pattern on FNAC including US, core-needle biopsy and elastosonography. Unfortunately, none of these has proved to be adequate to safely rule out the need for diagnostic surgery in follicular neoplasms [5–7].

 Thyroid scintigraphy with ^123^I-iodide or ^99m^Tc-pertechnetate is a well-established tool for ruling out malignancy in hyperfunctioning (i.e. hot) nodules, while thyroid ^99m^Tc-methoxyisobutylisonitrile (^99m^Tc-MIBI) scanning has been proposed for differentiating benign from malignant hypofunctioning (i.e. cold) thyroid nodules [1]. ^99m^Tc-MIBI uptake within the nodule reflects the abundance of actively functioning mitochondria in the nodule and therefore its oxidative burden. In turn, increased uptake and late retention of the tracer within the nodule indicate a high probability of a malignant thyroid nodule [1, 8]. Accordingly, many studies and a recent meta-analysis have demonstrated that a ^99m^Tc-MIBI scan is a sensitive diagnostic tool for predicting or excluding malignancy of hypofunctioning thyroid nodules, thus leading to a lower rate of unnecessary thyroidectomies [9–16].

 More recently attempts to refine the cytological diagnosis of follicular neoplasms have also involved the molecular testing of thyroid FNAC samples. Two main types of molecular test are currently available: mutation analysis panels and Afirma® gene expression classifier (GEC). The mutation analysis panels determine the presence of single gene point mutations (i.e. *BRAF*, *RAS*) or gene translocations (i.e. RET/PTC, PAX8/PPARγ). It has been estimated that one of these mutations is present in approximately 70 % of differentiated thyroid cancers (DTC); consequently nodules harbouring these mutations or translocations have a high likelihood of cancer, giving this test a high positive predictive value (PPV) [17]. However, a significant number (6 – 28 %) of malignant nodules do not harbour one of these genetic markers and, additionally, *RAS* mutations can be commonly found in benign adenomas [18]. The Afirma test employs a proprietary GEC. After RNA extraction and nucleic acid amplification, processed GEC samples are hybridized to a custom Afirma thyroid microarray and analysed with a classification algorithm using linear support vector machine logic to produce either a “benign” or “suspicious” test result. Validation studies have demonstrated a high negative predictive value (NPV) among nodules with indeterminate cytology [19] and the Afirma GEC has been reported to obviate the need for surgical excision in about 50 % of cases [20]. However, a substantial number of benign nodules do not have a gene expression profile classified as benign and the categorization of a nodule as “suspicious” carries a cancer risk of only 38 % [18].

 It is unclear whether molecular imaging or molecular testing is better for the identification of malignant follicular lesions. The aim of the present study was to prospectively investigate and compare the clinical performance of the mutation analysis panel and ^99m^Tc-MIBI thyroid scintigraphy in differentiating benign from malignant thyroid nodules in patients with a cytological reading of (suspicious for) follicular neoplasm.

## Materials and methods

### Patients

All patients were diagnosed and treated at the Division of Nuclear Medicine and Thyroid Centre, Oncology Institute of Southern Switzerland, Bellinzona (Switzerland) between 1 January 2010 and 31 December 2014. Enrolled were adult patients (i.e. age ≥18 years) with: (1) normal thyrotropin (TSH) and calcitonin levels; (2) a solitary hypofunctioning, thyroid nodule ≥10 mm in maximum diameter, with a suspicious US pattern; and (3) a cytological diagnosis of (suspicious for) “follicular neoplasm”. Mutation analysis on FNAC specimens and ^99m^Tc-MIBI scintigraphy were performed, and patients were subsequently operated on to confirm or exclude a malignant lesion. The study was approved by the Oncology Institute of Southern Switzerland Scientific Advisory Board and by the Ethics Committee of Canton Ticino (Bellinzona, Switzerland), and written informed consent was obtained from each patient.

### TSH and calcitonin measurement

TSH and calcitonin were measured using an IMMULITE® 2000 XPi platform (Siemens Healthcare Diagnostics, Erlangen, Germany). The normal limits were 0.40 – 4.00 mUI/L for TSH, and <8.4 ng/L (in men) and <5 ng/L (in women) for calcitonin.

### Thyroid ultrasonography

All US examinations were performed by the same physician (L.G.) with more than 20 years experience in thyroid US. An Acuson® S3000 sonograph equipped with a small-parts multifrequency (5 – 13 MHz) probe (Siemens, Erlangen, Germany) was used. Thyroid nodules were assessed according to the thyroid imaging reporting and data system (TI-RADS) classification [21] (i.e. *1* normal, *2* benign, *3* probably benign, *4A* undetermined pattern, *4B* suspicious pattern, *5* consistent with malignancy), and nodules with a score of 4B or 5 were referred for US-guided fine-needle aspiration.

### ^99m^Tc-Pertechnetate thyroid scintigraphy

^99m^Tc-Pertechnetate scintigraphy was performed using a large field-of-view γ-camera (Symbia-T2; Siemens, Erlangen, Germany) equipped with an ultrahigh-resolution parallel-hole low-energy collimator. Freshly eluted ^99m^Tc pertechnetate (74 MBq; Mallinkrodt, Petten, The Netherlands) was injected intravenously, and after 15 min a static image of the neck was acquired in the anterior view with a 128 × 128 matrix, using a digital zoom of 2 (pixel dimension 2.4 mm). The acquisition time was set to 300 s with a 20 % window centred at 140 keV in all cases. Thyroid scans were evaluated by an experienced nuclear medicine physician with more than 20 years experience in the field (L.G.) and nodules were assessed as hyperfunctioning (uptake in the nodule > uptake in normal thyroid tissue), functionally normal (uptake in the nodule = uptake of normal thyroid tissue) or hypofunctioning (uptake in the nodule < uptake in normal thyroid tissue.

### ^99m^Tc-MIBI thyroid scintigraphy

The tracer ^99m^Tc-MIBI was prepared using a commercially available kit (STAMICIS®; IBA, Gif-sur-Yvette, France) using freshly eluted^99m^Tc pertechnetate (see above). Labelling efficiency was assessed by thin-layer chromatography and was found to always exceed 95 %. ^99m^Tc-MIBI (200 MBq) was injected intravenously, and after 10 and 60 min static images of the neck were acquired in the anterior view with a 128 × 128 matrix, using a digital zoom of 2 (pixel dimension 2.4 mm). The acquisition time was set to 600 s with a 20 % window centred at 140 keV in all cases.

### ^99m^Tc-MIBI scan assessment

Before surgery images were independently assessed by two nuclear medicine physicians with more than 20 years experience in the field (L.C., A.C.). They used both a visual scoring method and a semiquantitative technique, and were not aware of the clinical data or the results of cytological and molecular analysis. If they disagreed, they discussed the case and reached a consensus in all cases.

#### Visual analysis

**Fig. 1:**
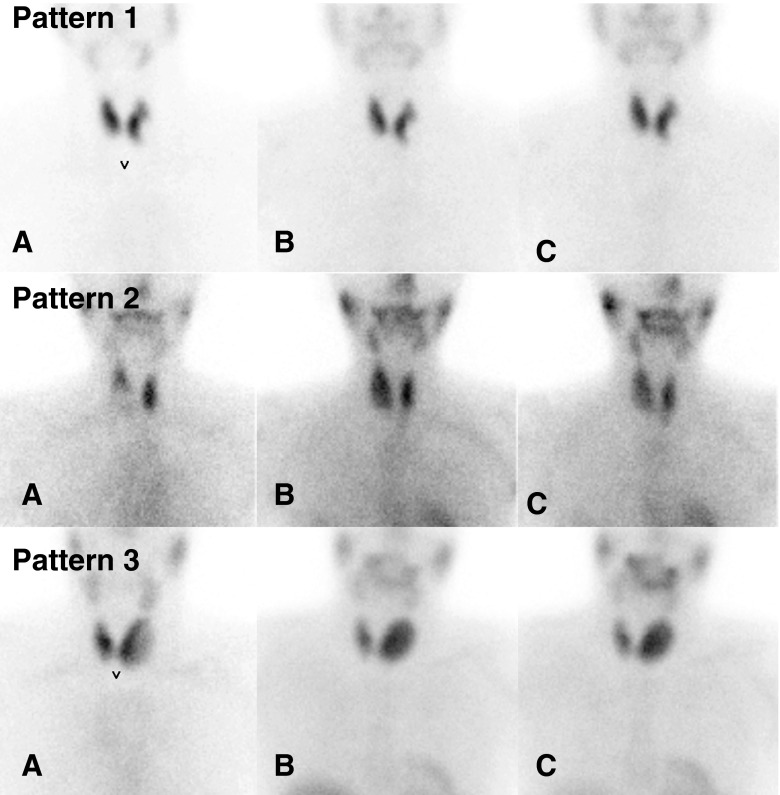
Visual assessment of ^99m^Tc-MIBI scintigraphy. **a** ^99m^Tc-pertechnetate; **b** ^99m^Tc-MIBI (early image); **c** ^99m^Tc-MIBI (delayed image) *Pattern 1* no early/delayed uptake within the nodule, *pattern 2* early uptake with late decrease *pattern 3* early uptake stable or increasing on delayed images

The ^99m^Tc-pertechnetate and ^99m^Tc-MIBI (early and delayed) thyroid images of a given patient were placed side by side for comparison. The visual assessment used the following scoring system: *pattern 1* no increased uptake of ^99m^Tc-MIBI within the nodule in comparison to the ^99m^Tc-pertechnetate scan on either the early or delayed image, *pattern 2* increased uptake on the early image that had decreased on the delayed image, and *pattern 3* increased uptake on the early image that remained unchanged or had further increased on the delayed image. Pattern 3 was considered suspicious for malignancy and patterns 1 and 2 were both considered negative for malignancy of the thyroid nodule concerned. The different scintigraphic patterns are illustrated in Fig. 1.

#### Semiquantitative analysis

On each early image, a region of interest (ROI) was drawn over the perimeter of the thyroid nodule and then moved to the opposite normal lobe; additionally, background ROIs were drawn above the inferior pole of thyroid lobe. These ROIs were then copied from the early to the delayed images. The mean count in each ROI was determined, and the early and delayed uptake results (EUR and DUR) were calculated by subtracting the count in normal tissue (after correction for background activity) from the nodule count. The washout index (WO_Ind_) was then calculated using the formula WO_Ind_ = [(DUR/EUR × 100)  − 100].

### Fine-needle aspiration cytology

Preoperative FNAC was performed under ultrasound guidance with a 22-gauge needle attached to a 10-mL plastic syringe by a nuclear medicine physician (L.G.) and a cytopathologist (M.B.) both with more than 20 years experience in fine-needle aspiration procedures. Some of the aspirated fluid was expelled and smeared onto charged slides, alcohol-fixed and processed for Papanicolaou staining, or air-dried and processed for Giemsa staining. The remaining material was fixed in PreservCyt solution and used to prepare liquid-based cytological slides and in some cases to prepare an additional Cytoblock cell block. Oncocytic follicular neoplasms were considered to be present if at least 50 % of the total follicular cells were large cells with abundant deeply eosinophilic and granular cytoplasm and hyperchromatic nuclei with prominent nucleoli. The same cytopathologist (M.B.) evaluated all cytological samples and the results were scored according to the Bethesda system [22]: *class I* nondiagnostic, *class II* benign, *class III* indeterminate/atypical, *class IV* (suspicious for) follicular neoplasm, *class V* suspicious for malignancy, and *class VI* malignant. Patients with a cytological reading of (suspicious for) follicular neoplasm (class IV) were enrolled in the present study.

### Mutation analysis

#### DNA/RNA isolation

Molecular tests were performed using laser capture microdissection (LCM) as previously described [23]. Briefly, one representative slide for each FNAC sample was incubated in xylene for 1 – 5 days to remove the cover slips and then processed for LCM using a laser microdissection system (PALM MicroBeam; Carl Zeiss Microscopy GmbH, Germany). For DNA analysis at least six microfollicular structures were dissected and catapulted in a tube cup containing 15 μl of ATL tissue lysis buffer (QIAamp mini kit; Qiagen, Chatsworth, CA). For RNA analysis, six additional microfollicular structures from the same slide were dissected and catapulted in a second tube cap containing 15 μl of RLT buffer (RNeasy micro kit; Qiagen). Genomic DNA was then extracted using the QIAamp mini kit according to the manufacturer’s instructions. RNA was extracted using the RNeasy micro kit. cDNA was prepared starting from 7.5 μL of RNA with SuperScript II reverse transcriptase (Invitrogen, Carlsbad, CA) according to the manufacturer’s instructions.

#### Genetic analyses

Mutations for *KRAS*, *HRAS* and *NRAS* (exons 2 and 3, containing hotspot codons 12, 13 and 61) and for *BRAF* (exon 15, containing the region surrounding the hotspot codon 600) were investigated by direct sequencing of genomic DNA and analysed with Sequence Navigator software (Thermo Fisher Applied Biosystems, Foster City, CA) as previously described [24, 25]. The PAX8/PPARγ fusion transcripts, the two main forms of RET/PTC translocations (RET/PTC1 and RET/PTC3), and the two internal control genes (PAX8 and PGK1) were analysed by reverse transcription polymerase chain reaction (RT-PCR) and the fragments were subsequently analysed using an ABI PRISM 3130 genetic analyser (Thermo Fisher) according to previously published protocols [26, 27], and then analysed with GeneMapper® software 5 (Thermo Fisher). All mutations or gene translocations were confirmed at least twice starting from independent PCR reactions. Synthetic DNA containing genomic regions involved in PAX8/PPARγ translocations as well as plasmids containing RET/PTC1 and RET/PTC3 fusion genes were used as positive controls.

### Statistical analysis

Histological diagnosis on the surgical specimens was regarded as the reference standard. The Mann-Whitney *U* test was used to evaluate the differences in EUR, DUR and WO_Ind_ between benign and malignant lesions. Receiver operating characteristic analysis was performed to determine the WO_Ind_ threshold above which malignant thyroid nodules could be detected. The results were considered significant when the *p* value was less than 0.05. Sensitivity, specificity, PPV, NPV, likelihood ratio, and diagnostic accuracy of mutational analysis and visual and semiquantitative ^99m^Tc-MIBI analyses were computed for benign versus malignant lesions. Statistical analyses were performed using Stats Direct version 2.8 (StatsDirect Ltd, Altrincham, UK)

## Results

Among 1,478 patients referred to our centre for thyroid nodule evaluation between 1 January 2010 and 31 December 2014 included were 61 consecutive patients (17 men and 44 women; age range 24 – 81 years, median 55 years) with a solitary thyroid nodule of at least 10 mm in diameter (median 22 mm, range 10 – 42 mm), hypofunctioning on ^99m^Tc-pertechnetate scan, suspicious on US examination. and cytologically classified as “(suspicious for) follicular neoplasm” (i.e. Bethesda class IV). Demographic and clinical data, cytopathology findings and the results of mutation analysis and ^99m^Tc-MIBI scintigraphy are summarized in Table 1. Overall, 16 (26 %) of the 61 nodules were malignant (5 follicular carcinomas; 5 follicular carcinomas, Hürthle cell variant; 3 papillary carcinomas; 2 papillary carcinomas, follicular variant; 1 poorly differentiated thyroid carcinoma) and 45 (74 %) were benign (37 follicular adenomas; 3 follicular adenomas, Hürthle cell variant; 5 hyperplastic nodules).

**Table 1:** Demographic, clinical, pathological and scintigraphic data of the enrolled patients

Patient^a^	Gender	Age (years)	Nodule size (mm)	TI-RADS class^b^	Bethesda class^c^	Histology	Visual analysis pattern^d^	Washout index	Translocations		Mutations					
									PAX8/PPARγ	RET/PTC	*BRAF*	*KRAS*	*NRAS*		*HRAS*	
												Exons 2 and 3	Exon 2	Exon 3	Exon 2	Exon 3
1	M	56	24	4B	IV	FTC	3	−6.5	Negative	Negative	WT	WT	WT	WT	WT	WT
2	F	71	28	4B	IV	FTC	3	−5	Positive	Negative	WT	WT	WT	WT	WT	WT
3	M	42	18	4B	IV	FTC	3	0	Negative	Negative	WT	WT	WT	WT	WT	WT
4	M	35	26	4B	IV Hürthle cell	FTC Hürthle cell	3	−6	Negative	Negative	WT	WT	WT	WT	WT	WT
5	F	61	45	4B	IV Hürthle cell	FTC Hürthle cell	3	−4	Negative	Negative	WT	WT	WT	WT	WT	WT
6	F	22	12	5	IV	PTC	2	−7	Negative	Negative	WT	WT	WT	WT	WT	WT
7	F	43	44	4B	IV Hürthle cell	FTC Hürthle cell	2	−4	Negative	Negative	WT	WT	WT	WT	WT	WT
8	M	57	31	4B	III	FTC	3	−8	Negative	Negative	WT	WT	WT	Q61R	WT	WT
9	F	34	36	4B	IV Hürthle cell	FTC Hürthle cell	3	−2	Negative	Negative	WT	WT	WT	WT	WT	WT
10	F	59	25	5	IV	PDTC	3	−2	Negative	Negative	WT	WT	WT	Q61R	WT	WT
11	M	58	50	4B	IV Hürthle cell	FTC Hürthle cell	2	−9	Negative	Negative	WT	WT	WT	WT	WT	WT
12	F	47	15	5	IV	PTC-FV	2	−7.2	Negative	Negative	WT	WT	WT	WT	WT	WT
13	M	65	21	4B	IV	FTC	2	−4.1	Negative	Negative	WT	WT	WT	WT	WT	WT
14	M	22	11	4B	IV	PTC-FV	3	−4	Negative	Negative	WT	WT	WT	WT	WT	WT
15	F	36	25	4B	IV	PTC	2	−7	Negative	Negative	WT	WT	WT	WT	WT	WT
16	M	59	12	5	IV	PTC	3	−2	Negative	Negative	WT	WT	WT	Q61R	WT	WT
17	F	29	22	5	IV Hürthle cell	AF Hürthle cell	3	−6.5	Negative	Negative	WT	WT	WT	WT	WT	WT
18	M	43	20	4B	IV	AF	2	−22	Negative	Negative	WT	WT	WT	WT	WT	WT
19	F	32	32	4B	IV	Hyperplasia	2	−18	Negative	Negative	WT	WT	WT	WT	WT	WT
20	M	67	19	4B	IV	AF	2	−22	Negative	Negative	WT	WT	WT	WT	WT	WT
21	F	51	27	4B	IV	AF	1	−26	Negative	Negative	WT	WT	WT	Q61R	WT	WT
22	F	55	12	4B	IV	AF	2	−23	Negative	Negative	WT	WT	WT	WT	WT	WT
23	F	29	18	4B	IV	AF	1	−20	Negative	Negative	WT	WT	WT	WT	WT	WT
24	F	48	43	4B	IV	AF	2	−18	Negative	Negative	WT	WT	WT	WT	WT	WT
25	M	37	22	4B	IV	AF	1	−26	Negative	Negative	WT	WT	WT	WT	WT	WT
26	F	38	44	4B	IV	AF	2	−18	Negative	Negative	WT	NV	WT	WT	NV	NV
27	M	27	25	4B	IV	AF	2	−24	Negative	Negative	WT	WT	WT	WT	WT	WT
28	F	46	32	4B	IV	AF	2	−20	Negative	Negative	WT	WT	WT	WT	WT	WT
29	F	33	17	4B	IV	Hyperplasia	1	−19	Negative	Negative	WT	WT	WT	WT	WT	WT
30	F	46	46	4B	IV	AF	2	−22	Negative	Negative	WT	WT	WT	WT	NV	WT
31	F	69	12	4B	IV	AF	2	−25	Negative	Negative	WT	WT	WT	WT	WT	WT
32	F	53	24	4B	IV	AF	2	−19	Positive	Negative	WT	WT	WT	WT	NV	NV
33	F	49	11	4B	IV	AF	2	−16	Negative	Negative	WT	WT	WT	WT	WT	WT
34	F	62	32	4B	IV	AF	2	−18	Negative	Negative	WT	WT	WT	WT	WT	WT
35	F	48	41	4B	IV	AF	1	−25	Negative	Negative	WT	WT	WT	WT	WT	WT
36	F	34	23	4B	IV	AF	2	−27	Negative	Negative	WT	WT	WT	WT	WT	WT
37	F	57	18	4B	IV	AF	2	−25	Positive	Negative	WT	WT	WT	WT	WT	WT
38	F	28	15	4B	IV	AF	2	−22	Negative	Negative	WT	WT	WT	WT	WT	Q61R
39	F	81	31	4B	IV Hürthle cell	AF Hürthle cell	3	−8	Negative	Negative	WT	WT	WT	WT	WT	WT
40	F	53	18	4B	IV Hürthle cell	AF Hürthle cell	2	−14	Negative	Negative	WT	WT	WT	WT	WT	WT
41	F	49	12	4B	IV	AF	2	−26	Negative	Negative	WT	WT	WT	WT	WT	WT
42	F	35	32	4B	IV	AF	2	−12	Negative	Negative	WT	WT	WT	WT	WT	WT
43	F	43	20	4B	IV	Hyperplasia	1	−27	Negative	Negative	WT	WT	WT	WT	WT	WT
44	F	27	30	4B	IV	Hyperplasia	1	−19	Negative	Negative	WT	WT	WT	WT	WT	WT
45	F	64	18	4B	IV	AF	2	−20	Negative	Negative	WT	WT	WT	WT	WT	WT
46	F	43	27	4B	IV	AF	2	−20	Negative	Negative	WT	WT	WT	WT	WT	WT
47	M	39	15	4B	IV	AF	1	−19	Negative	Negative	WT	WT	WT	WT	WT	WT
48	M	67	11	4B	IV	AF	2	−22	Negative	Negative	WT	WT	WT	WT	WT	WT
49	F	46	22	4B	IV	AF	2	−25	Negative	Negative	WT	WT	WT	WT	WT	WT
50	M	33	32	5	IV	AF	2	−24	Negative	Negative	WT	WT	WT	WT	WT	WT
51	F	41	14	4B	IV	AF	1	−18	Negative	Negative	WT	WT	WT	WT	WT	WT
52	F	52	28	4B	IV	AF	2	−20	Negative	Negative	WT	WT	WT	WT	WT	WT
53	F	55	22	4B	IV	Hyperplasia	1	−19	Negative	Negative	WT	WT	WT	WT	WT	WT
54	F	31	43	4B	IV	AF	2	−22	Negative	Negative	WT	WT	WT	WT	WT	WT
55	F	62	32	4B	IV	AF	2	−25	Negative	Negative	WT	WT	WT	WT	WT	WT
56	F	19	19	4B	IV	AF	1	−18	Negative	Negative	WT	WT	WT	WT	WT	WT
57	F	43	48	4B	IV	AF	2	−24	Negative	Negative	WT	WT	WT	WT	WT	WT
58	M	27	24	5	IV	AF	2	−20	Negative	Negative	WT	WT	WT	WT	WT	WT
59	F	42	17	4B	IV	AF	1	−25	Negative	Negative	WT	WT	WT	WT	WT	WT
60	F	57	21	4B	IV	AF	2	−22	Negative	Negative	WT	WT	WT	WT	WT	WT
61	M	40	32	4B	IV	AF	2	−20	Negative	Negative	WT	WT	WT	WT	WT	WT

*FTC* follicular thyroid carcinoma, *PTC* papillary thyroid carcinoma, *PDTC* poorly differentiated thyroid carcinoma, *FV* follicular variant, *WT* wild type, *NV* novel variant

^a^In patients 1–16 the nodules were malignant

^b^TI-RADS classification: *1* normal, *2* benign, *3* probably benign, *4A* undetermined pattern, *4B* suspicious pattern, *5* consistent with malignancy

^c^FNAC cytological samples scored according to the Bethesda system: *class I* nondiagnostic, *class II* benign, *class III* indeterminate/atypical, *class IV* (suspicious for) follicular neoplasm, *class V* suspicious for malignancy, *class VI* malignant

^d^MIBI thyroid images scored visually: *pattern 1* no increased uptake of ^99m^Tc-MIBI within the nodule in comparison to the ^99m^Tc-pertechnetate scan on either the early or delayed image, *pattern 2* increased uptake on the early image that had decreased on the delayed image, *pattern 3* increased uptake on the early image that remained unchanged or had further increased on the delayed image

### Mutation analysis

Neither *BRAF* mutations nor RET/PTC translocations were found in our series. PAX8/PPARγ mutations were found in one follicular carcinoma and two follicular adenomas, respectively. *RAS* mutations were found in three malignant nodules (one follicular, one papillary and one poorly differentiated carcinoma) and one follicular adenoma. Overall, mutational analysis was positive in four malignant and three benign follicular proliferations (sensitivity 25 %, specificity 94 %, accuracy 80 %, PPV 57 %, NPV 77 %). The positive likelihood ratio was 1.74 (95 % CI 0.94 – 4.78).

### ^99m^Tc-MIBI scintigraphy

#### Visual analysis

Among 16 malignant nodules, 10 (62 %) showed scintigraphic pattern 3, and 6 (38 %) pattern 2. Among 45 benign nodules, 2 (4 %) showed pattern 3, 31 (69 %) pattern 2, and 12 (27 %) pattern 1. Considering patterns 1 and 2 as negative for malignancy, the sensitivity, specificity and accuracy of ^99m^Tc-MIBI visual analysis in differentiating benign from malignant nodules were 62 %, 95 % and 87 %, respectively. PPV and NPV were 83 % and 88 %, respectively. The positive likelihood ratio was 4.56 (95 % CI 1.94 – 9.54).

#### Semiquantitative analysis

The WO_Ind_ cut-off corresponding to the highest accuracy for discriminating between benign and malignant follicular proliferations was settled at −9. Among 18 nodules with a WO_Ind_ between 0 and −9, 16 had a histological result consistent with malignancy (i.e. true-positive) whereas 2 were histologically benign (i.e. true-negative). The remaining 43 lesions with a WO_Ind_ equal to or below this cut-off were histologically benign. For this threshold, the sensitivity, specificity and accuracy were 100 %, 96 % and 98 %, respectively. The PPV and NPV were 88 % and 100 %, respectively. The positive likelihood ratio was 12.35 (95 % CI 8.76 – 21.45). The area under the receiver operating characteristic curve was 0.980.

## Discussion

In our series, 26 % of nodules with a follicular pattern on cytology were malignant with a prevalence of follicular carcinomas. These results probably reflect an accurate cytological classification that avoided both the upstaging of atypical features (class III) and the downstaging of suspicious papillary cancers (class V) to class IV. In turn, most “follicular neoplasms” were confirmed to be follicular cancer or follicular adenomas. In this setting, the present study showed that molecular analysis for the presence of DTC-associated genetic mutations is poorly sensitive (i.e. only one in four malignant nodules showed almost one mutation/translocation) and not accurate enough to obviate the need for surgery in patients with negative results (i.e. NPV 77 %). Neither *BRAF* mutations nor RET/PTC translocations were found in our patients. This result may be partially explained by the low number of papillary carcinomas in our series. However, a recent meta-analysis of the literature [28] has confirmed the very limited role of *BRAF* mutation analysis in nodules with a follicular pattern on cytology. In addition, among seven nodules with PAX8/PPARγ mutations and/or *RAS* translocations, four were malignant and three benign, leading to a very poor sensitivity and NPV.

Interestingly, visual pattern 1 on ^99m^Tc-MIBI scintigraphy (i.e. MIBI uptake lower than or equal to Tc uptake within the nodule) had a very high NPV, correctly excluding malignancy in 100 % of patients. However, this pattern is not frequent in benign follicular proliferations (12 of 45 patients in our series). Furthermore, an overlap between carcinomas and benign lesion was observed in nodules with scintigraphic pattern 2 or 3, resulting in a suboptimal sensitivity and PPV for visual analysis as a whole. In contrast, the semiquantitative analysis of ^99m^Tc-MIBI scintigraphy showed a better diagnostic performance than visual analysis in correctly identifying thyroid nodules cytologically classified as “follicular neoplasm” as benign or malignant, respectively. Using this semiquantitative method with the thresholds described here, very high levels of sensitivity, specificity, PPV, NPV and accuracy can be achieved. Certainly the present results show that in patients with equivocal ^99m^Tc-MIBI scintigraphy (i.e. pattern 2 or 3), a semiquantitative analysis should be performed to achieve a diagnostically useful result. Our results are largely in line with those previously obtained by Saggiorato et al. [12] in a series of 51 patients with follicular proliferation.

The sensitivity, specificity, accuracy, PPV and NPV of ^99m^Tc-MIBI in differentiating benign from malignant non-oncocytic tumours were 73 %, 81 %, 78 %, 73 % and 81 % for visual analysis and 100 %, 90 %, 95 %, 88 % and 100 % for semiquantitative analysis. In contrast, the diagnostic performance of both visual and semiquantitative analysis dramatically worsened in oncocytic lesions. Indeed, the diagnostic accuracy of ^99m^Tc-MIBI visual analysis was only 33 %, and no significant differences in the semiquantitative indices were observed between malignant and benign oncocytic thyroid nodules.

As oncocytic cells (both benign and malignant) are rich in mitochondria, it can be expected that most benign oncocytic lesions will also be positive. In our series eight patients with oncocytic follicular proliferations (five malignant and three benign) were included; in contrast to the study by Saggiorato et al. [12], we were able to accurately discriminate benign from malignant lesions using semiquantitative analysis. It can therefore be surmised that either mitochondrial density or blood flow is greater in malignant that in benign oncocytic proliferations, or both. This better performance in our study may be due to the comparatively high proportion of patients with malignant oncocytic nodules in our series which may have positively affected specificity and PPV. The high incidence of malignant oncocytic nodules among the patients in our series (60 %) compared to the literature (30 %) is well explained by the size of these lesions. Three of five malignant oncocytic nodules were larger than 40 mm, which in itself would be associated with a risk of malignancy of about 80 %. However, considering the still low number of patients with oncocytic lesions, no definitive specific conclusions can be drawn from the present study. Therefore, based on the present and previous data , we advise caution in employing ^99m^Tc-MIBI scanning in lesions cytologically diagnosed as oncocytic follicular proliferations. Nevertheless, as reported by Boi et al. [29], a visually negative scan strongly supports the absence of malignant oncocytic proliferation. Finally, molecular testing of FNAC material for the presence of DTC-associated mutations/translocations was clearly inferior to ^99m^Tc-MIBI scanning in our series, and should therefore not be considered for clinical routine for identifying follicular neoplasms.

Some limitations of the present study should also be discussed. First, the semiquantitative analysis is at least partially an operator-dependent technique, especially with regard to the definition of the ROIs. Furthermore, this technique is partially dependent on the instruments used and the peculiarities of the local patient population. Therefore, each laboratory needs to optimize the protocol and to define its own WO_Ind_ cut-off. Second, these results were obtained in patients prospectively evaluated in a single referral centre and need to be confirmed on a larger, multicentre population before universal adoption of this technique can be considered. Third, no data are available in the literature on direct prospective comparisons between ^99m^Tc-MIBI scan and Afirma GEC. Heinzel et al. [30] recently compared the published results of Afirma and molecular imaging with ^99m^Tc-MIBI and found that the latter is superior both in terms of life-years gained and cost/effectiveness. However, head-to-head comparisons in the same patient population are needed to clarify this issue. Notwithstanding these limitations, our results suggest that semiquantitative ^99m^Tc-MIBI could be a highly accurate presurgical method for investigating follicular lesions, predicting the malignant behaviour of thyroid nodules that are indeterminately diagnosed as follicular proliferations on FNAC. The use of this technique has great potential to reduce the number of diagnostic thyroid surgical procedures performed in patients with follicular proliferations without a great risk of missing DTC. However, based on the present results semiquantitative analysis may be unnecessary if ^99m^Tc-MIBI scintigraphy is unequivocally negative, i.e. no mismatch compared to ^99m^Tc-pertechnetate scintigraphy.

### Conclusion

In patients with a thyroid nodule cytologically diagnosed as follicular proliferation, semiquantitative analysis of ^99m^Tc-MIBI scintigraphy should be the preferred method for differentiating benign from malignant nodules. It is superior to both purely visual interpretation of ^99m^Tc-MIBI scintigraphy and to molecular testing for the presence of DTC-associated mutations in FNAC sample material.
